# Reliability and validity of the Chinese version of the Medical Outcomes Study HIV Health Survey (MOS-HIV) in people living with HIV/AIDS (PLWHA) in China

**DOI:** 10.1371/journal.pone.0201177

**Published:** 2018-07-25

**Authors:** Jie Liu, Yaxin Zhu, Bo Qu

**Affiliations:** Department of Health Statistics, School of Public Health, China Medical University, Shenyang, Liaoning Province, P.R. China; Griffith University, AUSTRALIA

## Abstract

**Objective:**

The aim of the study was to assess the psychometric properties of the Medical Outcomes Study HIV Health Survey (MOS-HIV) in people living with HIV/AIDS (PLWHA) in mainland China.

**Methods:**

A cross-sectional survey was conducted in 646 PLWHA between May 2015 and March 2016 in Dalian, Ningbo, and Zhengzhou City, China. The MOS-HIV includes 35 items and measures 10 scales. These ten scales can be effectively calculated under two summary scale scores, the physical health score (PHS) and the mental health score (MHS), with the physical functioning, pain and role functioning scales contributing to the PHS, the mental health, health distress, quality of life and cognitive function scales contributing to the MHS, and the energy/fatigue, general health and social functioning contributing to both factors. Reliability was measured in terms of internal consistency and test-retest reliability. The internal consistency of the questionnaire was analyzed using Cronbach’s α coefficient, and test-retest reliability was assessed using Pearson’s correlation coefficient. Validity was analyzed via construct validity, convergent and discriminant validity, and known group validity. Confirmatory factor analyses (CFA) were used to test construct validity. A multiple-group CFA analysis was conducted to investigate whether the MOS-HIV measured the same constructs across gender groups.

**Results:**

The MOS-HIV questionnaire was reliable and valid. Reliability of the PHS and MHS scales was 0.87 and 0.89, respectively. While the Cronbach’s α coefficients for each of the dimensions were > 0.70. According to the results of the confirmatory factor analysis (CFA), the hypothesized model was acceptable. The instrument showed factorial invariance across gender groups. All correlation coefficients were greater than 0.40, with a range of 0.60–0.94. The correlation coefficients observed between items and other dimensions were lower than the coefficients for the correlations between items and hypothesized dimensions for all scales, suggesting good convergent and discriminant validity. Patients with CD4 counts >500 cells/mm^3^ demonstrated better QOL than those with lower CD4 counts on six scales and the PHS (p<0.05) and symptomatic respondents had significantly lower scores than asymptomatic respondents on all the scales except health transition scales (p<0.05) suggesting good known group validity.

**Conclusions:**

The results of this study provide evidence that the MOS-HIV may be an acceptable, valid and reliable instrument for evaluating QOL of PLWHA in mainland China.

## Introduction

In recent years, the number of new HIV infections per year and the number of PLWHA have continued to increase in China [1]. Data suggest that the number of newly diagnosed cases has increased rapidly each year from 20,450 in 2011 to 45,145 in 2014 [2,3]. By the end of 2014, 501,000 cases of PLWHA, including 296,000 people living with HIV and 205,000 AIDS patients, had been reported based on the China Information System for Disease Control and Prevention [4]. Since 2003, free highly active antiretroviral treatment (HAART) has been provided to patients who agree with the conditions of treatment as a response to the HIV/AIDS epidemic in China [5]. Advances in treatment have improved survival rates in HIV-infected individuals, and AIDS has been shifted from a fatal disease to a chronic illness [6]. The result is that persons living with HIV are more likely to experience deteriorating physical health and psychological stress [7,8]. An important goal in the treatment of HIV-infected patients is, therefore, the effective enhancement of the quality of life (QOL) [9].

The body of literature on the measurement of QOL of PLWHA is growing [10–17]. QOL measures have been used to assess the physical and mental conditions of PLWHA, evaluate the effectiveness of treatment and intervention programs, identify the need for health services improvements, and investigate factors predicting well-being in PLWHA. QOL measures have become increasingly important and are receiving increasing attention.

Several QOL instruments have been applied in the evaluation of HIV-infected patients [18–21]. Each questionnaire has a unique structure and advantages. Among four HIV-specific QOL instruments, MOS-HIV demonstrated more satisfactory results based on the evaluation criteria in the review by Davis and colleagues [22]. The MOS-HIV measures three domains (cognitive functioning, health distress and quality of life) hypothesized to be associated with the health deterioration associated with HIV disease that are not measured by the SF-36 which is the commonly used measure in a wide variety of patient populations [18]. The MOS-HIV allows for the calculation of both individual scale and summary scores that permit more specific identification to be derived regarding the domains of Health-related quality of life (HRQOL) that are affected by HIV infection, AIDS, and/or its treatment and has become a popular instrument for measuring HRQOL of PLWHA.

Due to its brevity and comprehensiveness, various versions of the MOS-HIV have been translated into different languages. Studies have demonstrated that the reliability and validity of the versions of the MOS-HIV that have been translated into different languages and adapted for different cultures remain good in the PLWHA [21, 23–25]. Relatively few studies have been conducted to evaluate the QOL of PLWHA living in mainland China using the MOS-HIV. Thus, in this study, our aim was to test the reliability and validity of the MOS-HIV questionnaire to provide preliminary information for potential applications of the MOS-HIV in Chinese PLWHA.

## Materials and methods

### Respondents and procedures

A cross-sectional study was conducted in three cities (Dalian, Ningbo, and Zhengzhou) located in Liaoning province, Henan province, and Zhejiang province, respectively. The inclusion criteria were as follows: aged 18 years or older, infected with HIV, be capable of reading Chinese. Respondents unable to complete an interview and respondents unable to provide consent were excluded. A total of 646 PLWHA were recruited from the local Centers for Disease Control and Prevention (CDC) and infectious diseases hospitals between May 2015 and March 2016. Written informed consent was obtained from each respondent before survey initiation. Participation in the study was completely voluntary. All of the respondents completed a self-report questionnaire, and after survey completion, 50 Yuan (equivalent to 10 US dollars) was given to respondents as compensation for their time.

The questionnaire included the background information (age, gender, marital status, education, monthly income, CD4 count, HIV-related symptoms) and a Chinese simplified version of MOS-HIV, which was translated by professor Fen Yang [26]. The MOS-HIV includes 35 items and measures 10 scales, including 8 multi-items (general health, physical function, role function, cognitive function, pain, mental health, energy/fatigue and health distress) and 2 single items (social function and quality of life). Additionally, there was a single item named health transition. These ten scales can be effectively calculated under two summary scale scores, the PHS and the MHS, with the physical functioning, pain and role functioning scales contributing to the PHS, the mental health, health distress, quality of life and cognitive function scales contributing to the MHS, and the energy/fatigue, general health and social functioning contributing to both factors [26,27]. The raw scores for each scale were transformed to a scale of 0–100, with higher scores indicating ‘better’ QOL [28].

After the respondents completed the questionnaires, specially trained personnel reviewed the questionnaires, determined if the respondents had provided any non-standard or ambiguous answers, and contacted the respondents for timely verification. The study protocol was approved by the bioethics advisory commission of China Medical University (2014[34]).

### Statistical analysis

Descriptive statistics such as the mean score, standard deviation (SD), range and percentage of respondents scoring the floor and ceiling possible scores were generated. The reliability of the MOS-HIV questionnaire was measured in terms of internal consistency, which was expressed as Cronbach’s α coefficient. Reliability was considered to be adequate if the α value was >0.7. Test-retest reliability was assessed using Pearson’s correlation coefficient to determine the consistency of the questionnaire when administered two different times. To evaluate this construct, 60 randomly selected study subjects completed the questionnaire again 2 weeks later. Validity was analyzed in terms of convergent validity, discriminant validity, construct validity and known group validity. Convergent validity was assessed by correlation coefficients between each item and each scale to which they belong (General health; Physical function; Role function; Cognitive function; Pain; Mental health; Energy/fatigue; Health distress). Convergent validity was considered good if the coefficient for the correlation between each item and its related scales was >0.4. To demonstrate discriminant validity, items should be more highly correlated with their hypothesized scales than with the scales measuring other concepts. Confirmatory factor analyses (CFA) were used to test construct validity. Model fit was evaluated by examining the comparative fit index (CFI), non-normed fit index (NNFI), adjusted goodness-of-fit index (AGFI), standardized root mean squared residual (SRMR), and root-mean-square error of approximation (RMSEA). Conventionally, the model fit is considered acceptable if the CFI, AGFI, and NNFI are each 0.90 or greater. The general cutoff points for the RMSEA index and the SRMR at which a factor model is considered acceptable are below 0.08[29]. A multiple-group CFA analysis was conducted to investigate whether the MOS-HIV measured the same constructs across gender groups. First, the configural invariance was assessed. Then metric invariance was assessed by examining if the factor loadings were the same across gender groups. Changes in CFI (ΔCFI ≤ 0.01) were used to demonstrate factorial invariance across groups [30,31]. Known group comparisons were performed by determining whether individual MOS-HIV scale scores could discriminate among respondents with different CD4 cell counts (below 200 cells/mm^3^, 200 to 500 cells/mm^3^, and 500 cells/mm^3^ and above) and HIV symptoms (symptomatic and asymptomatic) using one-way Analysis of variance (ANOVA) and Student’s t-test.

The data were analyzed using SPSS® version 16.0 (SPSS Inc., Chicago, IL, USA) for Windows. CFA was carried out using LISREL 8.7. A *P*-value of < 0.05 was considered statistically significant.

## Results

### Background characteristics

Overall, 635 respondents completed the questionnaire, for a response rate of 98.3%. The mean age was 39.2 ± 7.6 years, with a range of 33–64 years. Of the respondents, 447 (70.4%) were men. The respondents’ CD4 count levels were as follows: 94 (14.8%) were below 200 cells/mm^3^, 332 (52.3%) were 200 to 500 cells/mm^3^, and 209 (32.9%) were 500 cells/mm^3^ and above. 135 (21.3%) respondents had HIV-related symptoms (Table 1).

**Table 1:** Distribution of background characteristics.

Item	Number	%
Age (years)		
<20	15	2.4%
20–30	195	30.7%
30–40	158	24.9%
>40	267	42.0%
Gender		
Male	447	70.4%
Female	188	29.6%
Marital status		
Single	222	35.0%
Married	321	50.6%
Divorced or widowed	92	14.4%
Education level		
Junior high school education or lower	279	43.9%
Senior high school	116	18.3%
College education or greater	240	37.8%
Monthly income		
<2000 RMB	282	44.4%
2000–4000 RMB	246	38.7%
>4000 RMB	107	16.9%
CD4 count (cells/mm^3^)		
<200	94	14.8%
200–500	332	52.3%
>500	209	32.9%
HIV symptoms		
Symptomatic	135	21.3%
Asymptomatic	500	78.7%

### Distribution of scores

The mean scores ranged from 44.1 to 85.2. The floor effects were not significant, the maximum of which was 9.1% (role function), whereas significant ceiling effects were observed for role function (35.4%), social function (17.8%), and cognitive function (15.7%). The mean MHS score and PHS score were 44.1 (SD = 11.0) and 50.9 (SD = 8.4) with no ceiling or floor effects (Table 2).

**Table 2:** Distribution of MOS-HIV scale scores.

Scales	No. of items	Mean	SD	% Floor	%Ceiling
General health	5	54.0	19.9	0.8	0.9
Physical function	6	85.2	18.5	0.9	11.1
Role function	2	76.6	25.1	9.1	35.4
Social function	1	65.3	13.3	7.2	17.8
Cognitive function	4	68.6	26.7	1.1	15.7
Pain	2	81.0	20.6	0.5	1.8
Mental health	5	60.7	19.9	0.2	1.8
Energy/fatigue	4	57.7	20.1	0.6	3.0
Health distress	4	65.9	18.1	1.6	1.7
Quality of life	1	61.1	11.5	2.4	10.7
Health transition	1	55.3	15.1	4.1	9.8
Mental health scores	-	44.1	11.0	0.0	0.0
Physical health scores	-	50.9	8.4	0.0	0.0

### Reliability analysis

The internal consistency reliability of the questionnaire was good. Reliability of the PHS and MHS scales was 0.87 and 0.89, respectively. The Cronbach’s α coefficients for the eight multi-item scales ranged from 0.79 to 0.93. Three dimensions (social function, quality of life and health transition) could not be assessed because they each consisted of only one item. The correlations observed between the items indicated that the test-retest reliability was good and that r > 0.70 could be achieved in all the domains (p<0.05), demonstrating that the MOS-HIV questionnaire had relatively good stability. The differences between the mean values calculated for each scale after two rounds of measurements were not statistically significant (Table 3).

**Table 3:** Internal reliability and inter-correlations of the MOS-HIV scales.

Scale	Test-retest reliability *n* = 60	Cronbach’*α* coefficient *n* = 635				Inter-correlations of the MOS-HIV scales							
			GH	PF	RF	SF	CF	PN	MH	EF	HD	QOL	HT
GH	0.71*	0.79	1										
PF	0.82*	0.87	0.38	1									
RF	0.76*	0.80	0.38	0.45	1								
SF	-	-	0.31	0.26	0.24	1							
CF	0.82*	0.90	0.43	0.44	0.37	0.51	1						
PN	0.85*	0.88	0.52	0.37	0.50	0.31	0.48	1					
MH	0.79*	0.87	0.52	0.31	0.28	0.35	0.57	0.4	1				
EF	0.85*	0.80	0.61	0.37	0.37	0.33	0.48	0.45	0.51	1			
HD	0.90*	0.93	0.45	0.32	0.28	0.52	0.43	0.41	0.55	0.49	1		
QOL	-	-	0.50	0.23	0.31	0.25	0.39	0.46	0.46	0.43	0.42	1	
HT	-	-	0.28	0.11	0.12	0.22	0.24	0.25	0.27	0.28	0.24	0.42	1

GH general health, PF physical function, RF role function, SF social function, CF cognitive function, PN pain, MH mental health, EF energy/fatigue, HD health distress, QoL quality of life, HT health transition

*p<0.05

### Validity analysis

Construct validity was evaluated using confirmatory factor analyses. The results of the factor analysis indicated that when the two component summary scores (PHS and MHS) were extracted from those of the ten scales, physical functioning, pain and role functioning loaded most strongly onto PHS, while mental health, health distress, quality of life and cognitive functioning loaded most strongly onto MHS; energy/fatigue, general health and social functioning contributed to both summary scores (Table 4). The fit index values were as follows: *χ*^2^ = 418.42 (*df* = 31, p<0.05), RMSEA = 0.04, SRMR = 0.061, NNFI = 0.91, CFI = 0.97, AGFI = 0.93. According to the fit index values, the fit of the hypothesized model was acceptable. The structure of the MOS-HIV was tested across gender groups to evaluate factorial invariance. The results of configural invariance showed the invariance of the factor structure across gender groups: *χ*^2^ = 515.97 (p < 0.05), RMSEA = 0.041, and CFI = 0.968. The findings for metric invariance showed that the factor loadings were the same across gender groups: *χ*^2^ = 514.29 (p < 0.05), RMSEA = 0.042, and CFI = 0.969. The change of CFI was 0.001 which was lower than 0.01 suggesting that the Chinese version of the MOS-HIV showed factorial invariance for PLWHA across gender groups.

**Table 4:** Standardized estimates of factor loading for the hypothesized model.

Scales	PHS	MHS
General health	0.54	0.22
Physical function	0.77	
Role function	0.63	
Pain	0.62	
Social function	0.22	0.57
Mental health		0.73
Energy/fatigue	0.03	0.79
Health distress		0.76
Cognitive function		0.83
Quality of life		0.75

The convergent validity and item-discriminant validity of the MOS-HIV are shown in Table 5. The coefficients for correlations between items and the hypothesized scale were 0.60–0.94. The correlations were all greater than 0.40, indicating a ‘perfect’ success rate and a good convergent validity. In addition, an excellent success rate was also achieved in terms of the item-discriminant validity tests. The item-discriminant validity correlations ranged from 0.05 to 0.70. Items were found to be significantly more correlated with their hypothesized scales than with the scales measuring other concepts (Table 5).

**Table 5:** Convergent validity and discriminant validity of the MOS-HIV.

	Coefficient range		Success rate (%)	
Scale	Convergent validity ^a^	Discriminant validity^b^	Convergent validity tests (%)	Discriminant validity tests (%)
General health	0.63–0.73	0.09–0.51	100	100
Physical function	0.60–0.78	0.05–0.40	100	100
Role function	0.80–0.89	0.08–0.47	100	100
Social function	-	0.22–0.52	-	100
Cognitive function	0.84–0.92	0.18–0.70	100	100
Pain	0.87–0.88	0.20–0.49	100	100
Mental health	0.68–0.76	0.12–0.67	100	100
Energy/fatigue	0.63–0.72	0.10–0.60	100	100
Health distress	0.89–0.94	0.20–0.68	100	100
Quality of life	-	0.23–0.49	-	100
Health transition	-	0.11–0.42	-	100

a:Correlations between items and hypothesized scales after correction for overlap

b:Correlations between items and other scales

Respondents with CD4 counts of more than 500 cells/mm^3^ were found to have better QOL scores on six of the ten scales (general health scale, physical function scale, role function scale, cognitive function scale, pain scale, energy/fatigue scale) and one of the summary scores (PHS) than respondents with lower CD4 counts (those with CD4 counts of less than 200 cells/mm^3^ or 200–500 cells/mm^3^, p<0.05). Symptomatic respondents had significantly lower scores than asymptomatic respondents on all the scales except health transition scales (p<0.05). These results indicate that the instrument has good known group validity (Table 6 and Table 7).

**Table 6:** Known group validity of the MOS-HIV scales for different CD4 counts.

Scale	<200 cells/mm^3^ Mean (SD)	200–500 cells/mm^3^ Mean (SD)	>500 cells/mm^3^ Mean (SD)	F	P
General health	47.8(17.5)	54.2(19.7)	56.4(20.5)	6.186	0.002
Physical function	81.2(21.0)	84.5(18.2)	88.0(17.4)	4.977	0.007
Role function	68.1(39.4)	75.3(36.1)	82.5(30.1)	6.085	0.002
Social function	64.3(32.1)	66.0(32.6)	64.6(35.0)	0.159	0.853
Cognitive function	64.4(28.9)	67.6(26.9)	72.0(25.0)	3.158	0.043
Pain	78.0(23.8)	79.7(21.1)	84.6(17.7)	5.008	0.007
Mental health	59.6(19.5)	59.5(20.3)	63.3(19.2)	2.581	0.076
Energy/fatigue	55.3(19.5)	56.2(20.6)	61.2(19.1)	4.816	0.008
Health distress	63.8(29.5)	65.6(28.4)	67.3(27.0)	0.556	0.574
Quality of life	59.8(19.1)	60.1(22.4)	63.3(21.1)	1.596	0.204
Health transition	58.0(23.2)	54.4(26.8)	55.5(23.0)	0.736	0.479
Mental health scores	42.8(11.0)	43.6(11.2)	45.4(10.5)	2.509	0.082
Physical health scores	48.8(9.0)	50.6(8.4)	52.2(7.7)	5.902	0.003

**Table 7:** Known group validity of the MOS-HIV scales on the basis of HIV symptoms.

**Scale**	**Symptomatic**	**Asymptomatic**	**t**	**P**
general health	50.2(19.0)	55.0(20.0)	-2.496	0.013
physical function	79.3(22.6)	86.8(16.9)	-4.231	0.000
role function	62.2(41.6)	80.5(32.0)	-5.494	0.000
social function	59.4(32.0)	66.8(33.4)	-2.312	0.021
cognitive function	57.4(28.1)	71.6(25.5)	-5.626	0.000
pain	71.2(22.2)	83.7(19.3)	-6.549	0.000
mental health	56.4(18.2)	61.9(20.1)	-2.891	0.004
energy	51.0(19.3)	59.5(19.9)	-4.396	0.000
health distress	60.6(28.7)	67.3(27.8)	-2.497	0.013
quality of life	54.8(23.2)	62.8(20.8)	-3.863	0.000
health transition	53.1(28.0)	55.9(24.2)	-1.131	0.258
mental health scores	40.6(10.5)	45.0(10.9)	-4.234	0.000
physical health scores	47.0(9. 1)	51.9(7. 8)	-6.216	0.000

## Discussion

As a multidimensional assessment of physical, psychological, and social functions, the MOS-HIV is believed to be a good measure of an individual’s state of health; it has become increasingly important and has received increasing attention. The MOS-HIV instrument has been found to be suitable and appropriate to assess QOL of HIV-infected individuals in many countries. The instrument has been reported to have good psychometric properties [6,23–25,28,32]. In China, the MOS-HIV questionnaire was also used to assess the quality of life and related influencing factors of PLWHA in Zhejiang, Henan, Shanxi, Guangxi and Yunnan provinces which showed acceptable reliability and validity in general [33,34]. As identified in other similar international studies, the results of our study indicated that the MOS-HIV had acceptable reliability and validity for determining QOL of Chinese PLWHA.

The degree of internal consistency across the items was expressed via Cronbach’s α coefficients. Some studies have reported the identification of lower Cronbach’s α coefficients (below 0.70) on some scales of the MOS-HIV. Chariyalertsak et al. reported identifying Cronbach’s α coefficients greater than 0.7 for all scales of the MOS-HIV (0.77–0.90) except the physical function subscale (0.67) in HIV-infected homeless and marginally housed individuals [20]. Hsiung et al. found that the Cronbach’s α coefficients all ranged from 0.82 to 0.95 across the scales of the MOS-HIV in patients with HIV infection except the coefficient for the role function subscale (0.54) [35,36]. In our study, the Cronbach’s α values ranged from 0.79 to 0.93, which indicated that reliability of the MOS-HIV scales were generally good.

In light of distribution of the MOS-HIV scores, three scales (role function (35.4%), social function (17.8%), and cognitive function (15.7%)) showed moderate ceiling effects; similar effects have been previously reported [6, 37–39]. This phenomenon could be in part attributable to the presence of fewer items in the role function, social function, and cognitive function domains [6, 37–39]. The observed ceiling effects may also imply that these scales have weak differentiation capabilities; however, the internal consistency tests confirmed that the items were more highly correlated with their own scales than with others.

Previous studies conducted in PLWHA demonstrated the presence of good construct validity of MOS-HIV [36, 27, 40]. In our study, the results of the chi-square test (*χ*^2^ = 418.42 and *p*<0.05) indicated a lack of fit. Hsiung et al. suggested that there was a tendency for the chi-square test to be influenced by sample size. A larger sample size may well have resulted in the derivation of significant results and indicated a lack of fit [35]. However, the coefficients for the NNFI (0.91), AGFI (0.93), and CFI (0.97) incremental fit indices were all above 0.90, and the coefficients for the two absolute fit indices (SRMR (0.061) and RMSEA (0.04)) were below 0.08, all of which suggested that the model had acceptable fit, indicating good overall construct validity. The change of CFI was 0.001 which was lower than 0.01 suggested that the Chinese version of the MOS-HIV showed factorial invariance for PLWHA across gender groups. The coefficients for correlations between items and the hypothesized scale were all greater than 0.40, indicating a ‘perfect’ success rate. In addition, an excellent success rate was also demonstrated by the results of the item-discriminant validity tests. Our results showed that the convergent validity and discriminant validity measures for all scales were satisfactory, representing findings that were consistent with those of previous studies [6, 35–39].

Some studies have suggested that MOS-HIV scores were not associated with CD4 cell count [6, 39, 41–43]. In contrast, most studies have supported the validity of the MOS-HIV in capturing CD4 cell count differences [23, 27, 40, 41, 43–48]. Based on the results of previous studies, subjects with low CD4 cell count would be expected to score lower on the MOS-HIV scales. In our study, six of the ten scales (general health scale, physical function scale, role function scale, cognitive function scale, pain scale, energy/fatigue scale) and one of the summary scores (PHS) scores demonstrated the ability to discriminate between groups of respondents stratified by CD4 cell counts. Better scores were observed in PLWHA with higher CD4 cell counts. These findings provide further evidence of the good known group validity of the MOS-HIV questionnaire and suggest that it may be a practical tool for use in the monitoring of health status in Chinese PLWHA.

However, we acknowledge that there are some limitations to this study. Study participants were recruited from 3 cities of China, which may have, to some extent, limited the representation of this study sample. Thus, it may have been better to survey a larger sample of PLWHA to increase the generalizability of study results.

## Conclusion

The MOS-HIV demonstrated good reliability and acceptable validity in assessing the QOL of Chinese PLWHA. It may serve as a valuable tool in evaluating QOL of Chinese PLWHA.
